# Deafness Weakens Interareal Couplings in the Auditory Cortex

**DOI:** 10.3389/fnins.2020.625721

**Published:** 2021-01-21

**Authors:** Prasandhya Astagiri Yusuf, Peter Hubka, Jochen Tillein, Martin Vinck, Andrej Kral

**Affiliations:** ^1^Department of Medical Physics/Medical Technology Core Cluster IMERI, Faculty of Medicine, University of Indonesia, Jakarta, Indonesia; ^2^Institute of AudioNeuroTechnology, Hannover Medical School, Hanover, Germany; ^3^Department of Experimental Otology of the ENT Clinics, Hannover Medical School, Hanover, Germany; ^4^Department of Otorhinolaryngology, Goethe University, Frankfurt am Main, Germany; ^5^MedEL Company, Innsbruck, Austria; ^6^Ernst Strüngmann Institut for Neuroscience in Cooperation with Max Planck Society, Frankfurt, Germany; ^7^Donders Centre for Neuroscience, Radboud University, Department of Neuroinformatics, Nijmegen, Netherlands; ^8^Department of Biomedical Sciences, School of Medicine and Health Sciences, Macquarie University, Sydney, NSW, Australia

**Keywords:** congenital deafness, predictive coding, bottom-up, top–down, cochlear implant, synchronization

## Abstract

The function of the cerebral cortex essentially depends on the ability to form functional assemblies across different cortical areas serving different functions. Here we investigated how developmental hearing experience affects functional and effective interareal connectivity in the auditory cortex in an animal model with years-long and complete auditory deprivation (deafness) from birth, the congenitally deaf cat (CDC). Using intracortical multielectrode arrays, neuronal activity of adult hearing controls and CDCs was registered in the primary auditory cortex and the secondary posterior auditory field (PAF). Ongoing activity as well as responses to acoustic stimulation (in adult hearing controls) and electric stimulation applied via cochlear implants (in adult hearing controls and CDCs) were analyzed. As functional connectivity measures pairwise phase consistency and Granger causality were used. While the number of coupled sites was nearly identical between controls and CDCs, a reduced coupling strength between the primary and the higher order field was found in CDCs under auditory stimulation. Such stimulus-related decoupling was particularly pronounced in the alpha band and in top–down direction. Ongoing connectivity did not show such a decoupling. These findings suggest that developmental experience is essential for functional interareal interactions during sensory processing. The outcomes demonstrate that corticocortical couplings, particularly top-down connectivity, are compromised following congenital sensory deprivation.

## Introduction

The auditory cortex is composed of a number of cortical areas with different functional roles (Malhotra et al., 2004; Winer and Lee, 2007). Together, these areas form a functional unit that allows constructing and perceiving sensory objects (Kral and Sharma, 2012; Bizley and Cohen, 2013). Only limited information exists on how these areas interact during such processes (Valentine and Eggermont, 2001), and it remains unclear how this interaction develops after birth. While it has been demonstrated that developmental hearing experience shapes the functional properties of individual brain areas (e.g., Klinke et al., 1999; Chang and Merzenich, 2003; Fallon et al., 2009), the role of experience for integration of cortical areas into a functionally unified auditory cortex is unclear. Despite a lot of effort in investigation of brain connectome (defined as the totality of all connections of the brain), only rudimentary information exists on its developmental constraints.

The question of developmental auditory experience is of particular relevance given that cochlear implants (CIs) restore hearing in congenitally deaf children (Kral and O’Donoghue, 2010). Developmental absence of hearing is accompanied by severe deficits in stimulus feature perception if hearing is restored late in life (Busby and Clark, 1999; Wei et al., 2007). On the other hand, CIs can compensate the deficits and provide access to spoken language with remarkable outcomes if implantations are performed within an early critical period (Manrique et al., 1999; Ponton and Eggermont, 2001; Sharma et al., 2002, 2005; Niparko et al., 2010). Later implantations are typically not successful because, in addition to the loss of (high) juvenile plasticity, congenital deafness strongly interferes with cortical development (review in Kral and Sharma, 2012; Kral et al., 2019): it (i) leads to delays in functional synaptogenesis and augmentation of functional synaptic ‘pruning,’ (ii) reduces the computational power of cortical networks and (iii) yields abnormally functioning cortical microcircuits. Furthermore, some cortical areas undergo a cross-modal reorganization (Rauschecker, 1995).

Cats have 2 primary and 11 higher-order auditory cortical areas (Rouiller et al., 1991; Winer and Lee, 2007). The posterior auditory field (PAF) is one of the secondary auditory fields (Stecker et al., 2003; Lee and Middlebrooks, 2013). It is part of the “where” pathway as defined in cats, primates and humans (Rauschecker and Tian, 2000; Lomber and Malhotra, 2008). Both bottom–up (e.g., from primary field A1 to secondary field PAF) and top-down (e.g., from PAF to A1) information flow are involved in its function (review in Hackett, 2011). The absence of hearing from birth leads to cross-modal reorganization of PAF, which becomes responsible for supranormal peripheral visual localization in congenitally deaf cats (CDCs) (Lomber et al., 2010). Primary field A1, on the other hand, is not involved in visual or somatosensory reorganizations (Kral et al., 2003; Lomber et al., 2010). A plausible hypothesis is therefore that A1 and PAF show an interareal decoupling in congenital deafness (Kral and Sharma, 2012). Here we test this hypothesis.

Several measures of connectivity have been described (Friston, 2011; Avena-Koenigsberger et al., 2018):

1. Structural connectivity is provided by the anatomical presence of connections (fiber tracts) between the structures of interest. Structural connectivity is typically analyzed by tracer studies in animals or diffusor tensor imaging in humans.
2. Functional connectivity defines statistical dependence among remote physiological events, as frequently analyzed using amplitude correlations or phase coherence, the latter being less dependent on individual response properties. Effective connectivity defines the influence one neural system has on another, either at synaptic or at population level, and is directional. Directional measures such as Granger causality (GC) are used to quantify the effective connectivity.

Structural connectivity provides a scaffold for functional connectivity, but structural and functional connectivity correlate only weakly (Suárez et al., 2020) since functional connectivity additionally captures the dynamics of interactions over time, and involves synaptic efficacy and responsiveness of target structures to patterns stored in the network (Avena-Koenigsberger et al., 2018). Furthermore, functional connectivity may result from common inputs that direct structural connections do not reveal but are functionally relevant for processing (Suárez et al., 2020).

Since the structural connectivity between A1 and PAF is generally preserved in both directions in CDCs (Barone et al., 2013; Butler et al., 2017), the aim of the present study was to compare functional and effective connectivity between A1 and PAF in hearing and deaf cats.

An efficient way to quantify functional connectivity is using the proxy of synchronization of band-specific neuronal activity (Fries, 2005; Womelsdorf et al., 2007; Buzsáki, 2009). Local field potentials allow such analysis (Fontolan et al., 2014; Kornblith et al., 2016). In auditory and visual system, increased synchronization of activity in theta and gamma bands contributes to bottom–up interareal influences, while the increase in alpha and beta bands contribute to top–down influence (Fontolan et al., 2014; van Kerkoerle et al., 2014; Bastos et al., 2015; Michalareas et al., 2016). The influence of congenital deafness on such synchronization is unknown.

As a higher-mammal model of complete sensory deprivation, congenitally deaf (white) cats (CDCs) were used here (Kral and Lomber, 2015). The organization of the auditory cortex in CDCs has been defined functionally and anatomically, including detailed functional maps of fields A1 and the anatomically surrounding fields (e.g., Kral et al., 2006, 2009; Berger et al., 2017). Auditory responses in PAF of CDCs have been characterized previously, too (Yusuf et al., 2017). The present study takes advantage of these previous observations.

In the present study, we compare invasive cortical recordings with multielectrode arrays in three groups of animals: adult hearing cats stimulated acoustically (acoustic controls, ACs), adult CDCs stimulated electrically with CIs, and adult hearing cats likewise stimulated with CIs (electric controls, ECs) following acute destruction of hair cells to prevent electrophonic responses (Sato et al., 2016). These results in two possible comparisons: (i) Whereas CDC and EC receive the same stimulus, they differ in their developmental sensory experience. (ii) AC and EC differ in the stimulus but have the same developmental sensory experience and thus a “similar brain.” This latter comparison thus provides information on the influence of stimulus modality (acoustic vs. unknown electric) on the stimulus response.

Phase coherence measures and GC were used to quantify the connectivity strength and the directionality of A1 – PAF interaction in response to auditory stimulation. Phase coherence is independent of response power (amplitude). Using these connectivity measures we tested the hypotheses whether the artificial electric stimulus generates less interareal interaction than the known acoustic stimulus, and whether CDCs show fundamentally reduced interareal interaction as a consequence of the total absence of hearing.

## Materials and Methods

### Subjects

Fifteen cats, ten adult hearing cats (hearing controls) and five adult CDCs were used in the present study. The details of the experimental procedure were described in previous publications (e.g., Yusuf et al., 2017) and will be briefly recapitulated here. The CDCs were selected from a colony of deaf white cats on the basis of absence of auditory brainstem responses at 120 dB SPL in a hearing screening after birth (Heid et al., 1998; Kral and Lomber, 2015). Each animal’s hearing status was confirmed at the beginning of the acute experiments in all animals (for details, see e.g., Berger et al., 2017).

To activate the auditory system in CDCs, the auditory nerve was stimulated electrically using a custom-made CI. As a control for the deaf group, hearing animals were acutely deafened prior to cochlear implantation (intracochlear neomycin application) to prevent responses from healthy hair cells (known as electrophonic hearing, Sato et al., 2016, 2017). Additionally, acoustically stimulated hearing animals were included so that the natural connectivity elicited by acoustic stimuli could be investigated. Thus, the study included three animal groups: ACs (*n* = 6), ECs (*n* = 6), and CDCs (*n* = 5). Of the hearing animals, two were first stimulated acoustically and subsequently stimulated electrically in order to confirm, at the individual level, the effects observed in the group data. Consequently, while 6 animals were in both control groups, only 10 hearing cats were used in total.

The experiments were approved by the local state authorities and were performed in compliance with the Guidelines of the European Community for the care and use of laboratory animals (EUVD 86/609/EEC) and the German Animal Welfare Act (TierSchG).

### Experimental Procedures

All animals were premedicated with 0.25 mg atropine i.p. and initially anesthetized with ketamine hydrochloride (24.5 mg/kg, Ketavet, Parker-Davis, Germany) and propionyl promazine phosphate (2.1 mg/kg, Combelen, Bayer, Germany). They were then tracheotomized and artificially ventilated with 50% O_2_ and 50% N_2_O, with the addition of 0.2–1.5% concentration of isoflurane (Lilly, Germany) to maintain a controlled depth of anesthesia in desynchronized cortical state identified by suppression index values within between 1 and 3, by absence of burst-suppression periods and absence of spindles/bursting (Land et al., 2012). End-tidal CO_2_ was continuously monitored and maintained at 4%, and the core temperature was kept at 37.5 – 38.0°C using a homeothermic blanket connected to a rectal temperature probe. Monitoring of the animal’s status also involved blood gas concentration measurements, pH, bicarbonate concentration and base excess, glycemia and oxygen saturation determined in capillary blood. A modified Ringer’s solution containing bicarbonate and plasma expander was infused i.v. through a venous catheter to supply volume with additional bicarbonate depending on the acid-base status. Use of a higher mammal allows guaranteeing a constant (stable) overall condition of the animal by monitoring and correction of the acid-base balance performed every 12 h throughout the experiments (48–72 h). Furthermore, continuous monitoring of the electrocardiogram, electroencephalogram, breathing pressure and capnometry ensured optimal vital state throughout the whole experiment.

Following tracheotomy, placement of venous and urine catheter, and removal of both pinnae in order to directly access the tympanic membrane for closed-system acoustic stimulation, the animal’s head was fixed in a stereotactic frame (Horsley-Clarke). Both bullae and ear canals were subsequently exposed. To record auditory brainstem responses (ABRs), a small trephination was drilled at the vertex of the skull and a silver-ball electrode (diameter 1 mm) was attached epidurally. The indifferent electrode used for the recordings was inserted medially into the neck muscles.

Hearing status was verified using ABRs with 50 μs condensation clicks applied through a closed system directly to the tympanic membrane using a calibrated speaker (DT48, Bayer Dynamics, Germany) at levels up to 120 dB SPL. Brainstem evoked signals were recorded using an epidural vertex electrode against a reference at the midline of the neck, were preamplified (60 dB, Otoconsult V2 low-impedance amplifier), amplified at a second stage (40 dB, Otoconsult Amplifier-Filter F1, filters 0.010–10 kHz) and recorded using National Instruments MIO cards (National Instruments, Munich, Germany). The signals were averaged (200 sweeps, repetition rate 33 Hz, Audiology Lab, Otoconsult, Frankfurt am Main, Germany). Absence of acoustically evoked brainstem responses (including wave I, generated within the auditory nerve) to clicks above 120 dB SPL verified complete deafness. In hearing cats, the thresholds were less than 40 dB SPL before the animals were deafened by slow instillation of 300 μl of neomycin sulfate into the scala tympani (within 5 min.). The Neomycin was left in place for a further 5 min. and subsequently washed out by slow instillation of Ringer’s solution. Total absence of brainstem evoked responses verified that the deafening procedure was successful. For electrical stimulation, hearing cats and CDCs were implanted with a CI inserted via the round window. The implant consisted of a medical-grade silicone tube with five intrascalar contacts: a small golden sphere at the tip (diameter 0.8 mm) and four golden rings, the distance between all electrodes being 1 mm. The intrascalar part of the implant was tapered in the apical direction from a diameter of 1.6 mm to 0.8 mm. The extracochlear silicone tube had a diameter of 1.6 mm. The gold contacts were connected to a seven-strand Teflon-coated stainless-steel braided wire. The stimulation mode was wide bipolar (most apical vs. the fourth intracochlear electrode in the basal direction; distance between active electrodes was thus 3 mm).

Electrically evoked auditory brainstem response (E-ABR) to single biphasic pulses was recorded and the lowest current levels evoking a brainstem response (E-ABR-threshold currents) were determined. For this purpose, charge-balanced biphasic pulses (200 μs/phase, repetition rate 33 pps) were applied to the CI using wide bipolar stimulation (most apical and most basal electrode). Stimulation was performed with optically isolated current sources (CS1, Otoconsult, Frankfurt am Main, Germany).

### Stimulation and Recording

Trephination was performed above the auditory cortex and the dura was removed. The cortex was photographed to document the recording positions. Using an ORIEL motorized x-y-z micromanipulator (1 μm precision in all directions), a silver-ball macroelectrode (diameter 1 mm) was positioned at a regular raster of nine cortical positions on the primary auditory cortex (field A1). The dorsal end of the posterior ectosylvian sulcus was used as a reference point. Signals (local field potentials, LFPs) recorded in response to an electric biphasic pulse applied through a CI were preamplified (60 dB, Otoconsult V2 low-impedance amplifier), amplified at a second stage (20 dB, Otoconsult Amplifier-Filter F1, filters 0.010–10 kHz), recorded using MIO cards and averaged (100 sweeps, repetition rate 1.97 Hz). The signals were stored and threshold current levels were evaluated at all recording positions with a precision of ±1 dB.

In order to determine the extent of the cortical activated region, a Ringer-filled glass microelectrode (impedance < 6 MΩ) was used for mapping the field A1. LFPs on the cortical surface were recorded at 75–150 cortical positions during stimulation with the CI, using single biphasic pulses (200 μs/phase, wide bipolar stimulation at both the ipsilateral and contralateral ear, stimulation current 10 dB above the lowest cortical threshold determined using the macroelectrode). The stimuli were applied at a repetition rate of ∼2 pps. Recorded signals were bandpass filtered (10–9000 Hz) and amplified 5000 times (Neuralynx Cheetah, Bozeman, MT, United States). The data were digitized using a NI PCIe 6259 MIO card at a sampling rate of 25 kHz per channel. Fifty responses were averaged to obtain evoked LFPs. Amplitudes of these middle-latency responses (peak to baseline) were used to construct cortical activation maps and determine the most responsive region in A1, the “hot spots” (Kral et al., 2009).

**FIGURE 1:**
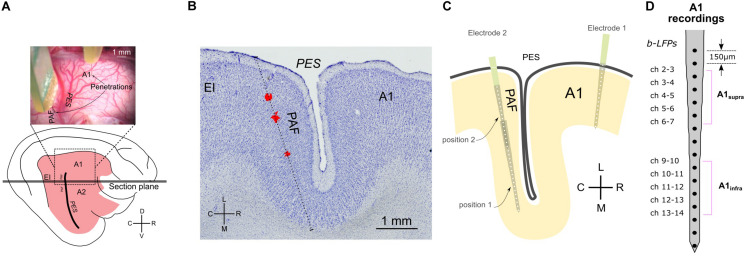
Methodology and recording positions. **(A)** Top: photograph of feline cortex after trephination, revealing the penetration sites in A1 and PAF. Bottom: illustration of entire brain from the same perspective. **(B)** Reconstruction of penetration of the DiI stained probe in a Nissl-stained section. The sectional plane is shown in **(A)**. The stained images were stacked and aligned to reconstruct the penetration. The red deposits shown (DiI) were extracted from several successive florescence images from the same region of the cortex and projected onto the Nissl-stained section. The reconstructed direction of penetration is shown as a dotted line. **(C)** Schematic illustration of electrode penetrations in A1 and PAF. In PAF, dense mapping allowed capture of auditory responses in each animal. Using two recording depths, each penetration includes 32 recording sites in total. **(D)** Channels of bipolar derivation LFP (b-LFP) in A1 recordings, grouped into supragranular (A1_supra_) and infragranular (A1_infra_) layers. A1, primary auditory cortex; EI, intermediate area of the posterior ectosylvian gyrus; PAF, posterior auditory field; PES, posterior ectosylvian sulcus; V, ventral; D, dorsal; R, rostral; C, caudal; L, lateral; M, medial.

Simultaneous recordings from the right A1 and PAF were performed contralateral to the stimulated ear. In A1, using a micromanipulator the cortex was penetrated perpendicular to the surface in the ‘hot spot’ (responses with >300 μV amplitude, Kral et al., 2009) with a single-shank Neuronexus probe (16 contacts, 150 μm spacing, around 1–2 MΩ impedance). The probe was inserted so that the last contact just disappeared into the cortex (penetration depth ∼2400 μm). Since PAF is hidden in a sulcus, the recording electrode could not be inserted radially as in A1. To cover the complete PAF, we recorded the LFP signals from two penetration depths (electrode tip depth at 5,000 and 2,500 μm penetration depth) using a second Neuronexus probe with the same characteristics as the first (Figure 1). This was performed through the dorsoventral extent of this field parallel to the course of the posterior ectosylvian sulcus with a penetration-to-penetration distance of ∼500 μm in the dorsoventral direction. All manipulation was performed using micromanipulators (precision ∼1 μm) and under visual control through the operating microscope (OPMI1-H, Zeiss Deutschland, Oberkochen, Germany). Recorded signals were bandpass filtered (1–9000 Hz) and amplified 5,000 times (Neuralynx Cheetah, Bozeman, MT, United States). The data were digitized using a NI PCIe 6259 MIO card at a sampling rate of 25 kHz per channel. During these recordings, the cortex was stabilized by means of a modified Davies chamber (Tillein et al., 2010). The reference for both probes was the vertex silver-ball electrode placed epidurally. Off-line, bipolar derivation of the signals in A1 before connectivity analysis ensured that the reference did not influence connectivity results.

The ACs were stimulated acoustically using three condensation clicks (50 μs duration at 500 pps) at different sound pressure levels. The ECs and CDCs were stimulated using a custom-made CI inserted into the scala tympani through the round window. The stimulus was a train of charge-balanced biphasic pulses (200 μs/phase, repetition rate 500 pps, three pulses in the train applied). Stimulation was in wide bipolar configuration. The acoustic and electric stimuli were applied at a repetition rate of 1/1537 ms, with 30 stimulus repetitions per condition (level). Stimulus increased in 10 dB increments in acoustic stimulation and in 1–2 dB increments in electric stimulation. Stimulus artifacts were removed by linear interpolation of the 6 ms period during stimulation. In a previous study we ensured that this did not introduce any artifacts into frequency-specific signals used (Yusuf et al., 2017).

The stimulation levels for connectivity analysis were chosen according to input–output level functions (levels that reach the saturation of evoked response). Analyses reported in the present study were performed at 40 dB (acoustic) above ABR threshold, while electrical stimulation was administered to ECs and CDCs using three biphasic electric charge-balanced pulses at 6 dB (electric) above the electrically evoked auditory brainstem response (E-ABR) threshold.

### Histology

For each animal, at least one penetration for each field was marked by a fluorescent dye (DiI, 1,10-dioctadecyl-3,3,3′,3′-tetramethylindocarbocyanine perchlorate; Invitrogen). Since the probe attachment to the stereotactic frame was constant throughout the experiment, it was possible to extrapolate all penetrations directions from the stained and reconstructed tract. In PAF, histological reconstructions confirmed the correct location within this field in all animals reported.

After the experiments, the animals were transcardially perfused in deep anesthesia. Following thoracotomy, 0.5 ml heparin (Heparin Natrium, Ratiopharm, Ulm, Germany) was injected into the both ventricles. Two liters of 0.9% NaCl solution and two liters of fixative (4% paraformaldehyde) and one liter of 10% sucrose were infused transcardially. The perfusion pressure was kept constant at 120–150 mmHg and monitored using the Perfusion One system (Leica Biosystems, Buffalo Grove, IL, United States). If required, the brain was postfixated in 4% paraformaldehyde and 10% sucrose overnight. For cryoprotection, each brain was placed in 30% sucrose solution until it sank. Subsequently, the brain was blocked, frozen at −80°C and cut at −20°C using a Leica Cryostat CM3050S (Leica Microsystems GmbH, Wetzlar, Germany) in section 50 μm thick. The sections were first photographed to reveal the DiI in fluorescent mode using a Keyence BZ-9000 microscope and subsequently stained using Nissl staining and SMI-32. For reconstruction, native fluorescence images were combined with the same Nissl-stained sections.

Layers in A1 were grouped into supragranular, granular and infragranular based on the reconstructions of penetrations. The Nissl staining reveals the border of layer IV to layer V (Berger et al., 2017). Additionally, current source density measures (CSDs) that show a typical sequence of middle source in layer III and deep sink in layer V, with an initial sink followed by a source in layer IV between them (Kral et al., 2006), confirm this differentiation.

### Time Domain Analysis

All data processing and analyses mentioned in this section were performed offline using the FieldTrip toolbox^1^ (Oostenveld et al., 2011) and custom-made MATLAB scripts (Mathworks Inc., Aachen, Germany). Occasional noisy recordings caused by unstable probe contacts, channels with artifacts and occasional trials with spindles were not included in the analyses.

Discrete Fourier transformation (DFT) filters at 50 and 100 Hz were applied to remove power line artifacts. The detrend (demean) procedure was applied to the LFP signals to remove any possible DC shift in the recordings. We reduced the far-field components in A1 by subtracting every two adjacent channels within an electrode shank from each other, yielding the bipolar derivation LFP (b-LFP) signals. We removed the transient evoked components by subtracting the time domain averaged signal from each trial, allowing the analysis of the non-phase-locked part only (Donner and Siegel, 2011; Siegel et al., 2012). In the following, all connectivity analyses were computed from the non-phase-locked signals of bipolar derivation LFPs in A1 and non-phase-locked unipolar LFPs in PAF.

### Spectrum Analyses

Hanning-tapered Fourier transformation was computed based on the LFP data in the prestimulus/baseline time window (−400 to −1 ms) and in the late-latency poststimulus time window (200–600 ms). Frequencies from 1 to 128 Hz with 1 Hz linear increments were subsequently analyzed. Power spectra were generated by taking the absolute square of the transformation.

Time-frequency representations (TFRs) were computed by means of complex wavelet analysis (using Morlet wavelet, *m* = 6) with 56 logarithmic frequency increments from 4 to 128 Hz, thus capturing the theta (4–8 Hz), alpha (8–16 Hz), beta (16–32 Hz), low-gamma (32–64 Hz), and high-gamma (64–128 Hz) frequency bands, in an equal number of bins (Hipp et al., 2011).

### Functional Connectivity

We computed the phase coherence between A1 and PAF electrodes using debiased weighted phase-lag index (Vinck et al., 2011) (WPLId) and pairwise phase consistency (Vinck et al., 2010) (PPC). These methods are insensitive to sample size bias (WPLId) or unbiased to sample size (PPC), which fits with the availability of 30 trials in this study. The values range from zero (negative values due to limited sampling were corrected to zero) to one (maximum coherence).

As WPLId includes only the imaginary part of the cross-spectrum, it is sensitive only to the true interaction between two signals but not to the common reference and far-field (volume conduction) signals (Vinck et al., 2011). A higher signal-to-noise ratio is also found in comparison with other connectivity measures based on the imaginary component of the cross-spectrum (Phillips et al., 2014; Babapoor-Farrokhran et al., 2017). Due to its sensitivity in detecting true interaction, here WPLId was used for defining *significant coupling*. The WPLId value was *z*-score normalized to its standard deviation (Nolte et al., 2008), estimated by the applying leave-one-out jackknife procedure (Richter et al., 2015) from the multiple observations (trials), as follows

(1) $$wPLId_{z}=\frac{wPLId}{std\left(wPLId\right)}$$

This enables phase coherence to be reliably indexed using z-scores. The *significantly coupled channel pairs* were computed by thresholding the couplings with maximum *z*-score values exceed the equivalent of *p* < 0.05 (Bonferroni corrected). Subsequently, we recomputed the functional connectivity using the PPC method, only including channel-pairs with significant coupling. PPC yields results proportional to true angle distribution and therefore we focused on this method (WPLId results are available and were consistent in outcome with PPC).

The PPC method computes the vector dot product (i.e., the projection of one vector onto another) for all given trial pairs of relative phases. The higher the phase consistency across trials, the smaller the angular distance, and hence the higher the dot products for each pair. The PPC value is defined as the average of the dot product across all available pairs [0.5 * N * (N-1), where *N* denotes number of trials] (Vinck et al., 2010). Unless specifically mentioned, all PPC values are presented in change to baseline, subtracting the late-latency poststimulus time PPC with the prestimulus time PPC.

### Effective Connectivity

Effective connectivity was computed using the non-parametric GC (Dhamala et al., 2008). GC analysis is useful for quantifying bidirectional interaction, i.e., separately quantifying GC influence from A1 to PAF (GC_A__1__→__PAF_) and the influence from PAF to A1 (GC_PAF__→__A__1_). GC spectra were obtained by computing Geweke’s frequency domain GC (Geweke, 1982) and the spectral factorization technique was used for complex cross-spectral density, obtained from the Fourier transformation. Non-parametric GC is advantageous since it does not require model order for autoregressive computation (as in the parametric GC), but has a drawback: cross-spectral density yields a smoothened shape (Bastos and Schoffelen, 2016). GC values are presented as change to baseline, subtracting the late-latency poststimulus time GC with the prestimulus time GC.

Directionality (GC_flow_) was computed as GC_A__1__→__PAF_ minus GC_PAF__→__A__1_. Consequently, positive values represent the domination of bottom-up interaction (A1→PAF) while negative values represent the domination of top–down interaction (PAF→A1).

We computed reversed-time GC to check for any false GC analysis results due to the presence of correlated and uncorrelated noise in the signal (Vinck et al., 2015). Time reversal of the signal prior to GC computation should consequently reverse the domination of directionality (GC_flow_). The presence of noise in the signal will not change this flow domination, i.e., from a positive to a negative, or from a negative to a positive value (Vinck et al., 2015). Therefore, time-reversing the signal is an effective procedure for confirming the directionality from GC analysis. We excluded channel pairs from the grand average computation where the requirement for ‘flipped directionality’ in the reversed-time GC was not satisfied.

### Statistics

We compared acoustic and ECs to reveal the influence of stimulation mode, and ECs with CDCs to reveal the effect of congenital sensory deprivation. ACs could not be directly compared with CDCs due to several biasing factors: they differed not only in developmental sensory experience but also in the mode of stimulation (acoustic vs. electric) and in the presence of hair cells generating spontaneous activity. Thus, differences would be equivocal with respect to several factors.

The differences between each pair of groups (CDCs vs. ECs and acoustic vs. electric controls) for the spectrum-based analyses were tested using the Wilcoxon rank-sum test, corrected with false discovery rate procedure (Benjamini and Yekutieli, 2001). For the TFR-based analyses, we used non-parametric cluster-based permutation statistics (Maris and Oostenveld, 2007) with 1,000 random permutations under the null hypothesis (cluster α threshold 0.5%, two-tail significant α value = 0.25%) – (i) compared against zeros for significant increase and decrease in each site-pair and group and (ii) compared between groups yielding pair comparison in each site-pair.

## Results

Local field potentials (LFPs) in primary auditory cortex (A1) and the posterior auditory field (PAF) were recorded in the cortex contralateral to the stimulated ear. The cortex was penetrated perpendicularly to the cortical surface at the most responsive area of A1 (the hot spot, same as in Tillein et al., 2010, 2016; Yusuf et al., 2017) with a multielectrode array. Recordings in PAF were performed throughout the entire dorsoventral extent of the field parallel to the posterior ectosylvian sulcus using another multielectrode array at up to 10 penetrations in PAF of each animal (Figure 1). This resulted in layer-specific recordings in A1 and tangential recording tracks in PAF (Figures 1B,C). To minimize the contribution of volume conduction effect on connectivity analysis between A1 and PAF, and to localize the sources of LFPs to individual layers, off-line signal subtraction between neighboring channels (bipolar derivation LFP) in A1 was calculated (Figure 1D). We determined the cortical depth of each channel and grouped them to the corresponding layers within A1 (Table 1, see Berger et al., 2017). In the following, we combined A1 recordings within supragranular layers and within infragranular layers (denoted as A1_supra_ and A1_infra_). Layer IV in A1 was excluded from the subsequent statistical analysis because long-range corticocortical connections are not present in layer IV of A1: its inputs originate in the thalamus (Mitani and Shimokouchi, 1985; Markov et al., 2014). In PAF, due to the tangential course of penetration, precise identification of recorded layers was not possible for all electrode contacts. The use of unipolar signals allowed additionally increasing the sensitivity for coupling by capturing signals from sources not directly within the penetration in PAF. Thus for determining the coupling we used local sources in A1 and less local sources in PAF.

**TABLE 1:** Cortical depths for each electrode of the probe in A1 over the range of deviations between 0° and 14^*o*^ from perpendicular as observed in the present experiments.

	Layer border [μm]	Unipolar		Bipolar	
		Channel #	Cortical depth [μm]	Channel #	Cortical depth [μm]
Supragranular	150–900	2 to 7	∼146–900	2–3 to 6–7	218–825
Granular	900–1150	8	1019–1050	7–8 to 8–9	946–1125
Infragranular	>1150	9 to 16	>1164	>9–10	>1237

*In total, we analyzed 470 sites in supragranular and 663 sites in infragranular layers of acoustic controls, 787 sites in supragranular and 711 in infragranular layers in electric controls, and 1147 sites in supragranular and 1342 sites in infragranular layers of CDCs.*

**FIGURE 2:**
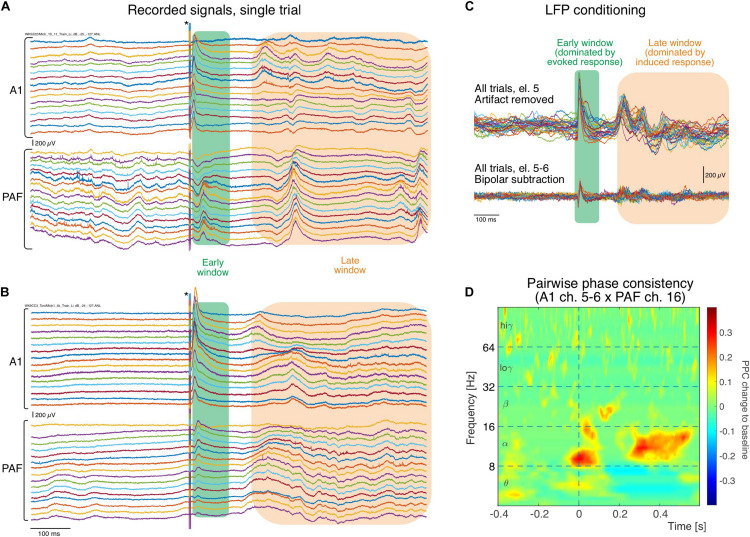
Single raw trace LFP examples recorded simultaneously in different cortical positions (in both fields arranged from surface to deep) during stimulation. **(A)** In an electric control, the electric stimulus generates a large artifact that is discernible in all recordings that lasts throughout the 6 ms of stimulus duration (asterisk). Following the stimulus a short latency response (green rectangle) is observed in all traces of recordings, larger in A1 and smaller in PAF. Two hundred ms after the stimulus, some increased activity can be observed that is less well synchronized to the stimulus than the early response (orange rectangle). **(B)** In the congenitally deaf cats, similar activity in both windows is observed in A1 and PAF (for systematic differences, see Yusuf et al., 2017). **(C)** Example of trial-to-trial variability for one electrode following stimulation artifact removal, obtained from the A1 recording shown in **(A)**. The early window response shows higher trial-to-trial consistency than the late response, corresponding to the previous description of an evoked response caused by thalamic input. In the late window the responses vary between trials in latency/phase and amplitude, typical for induced responses resulting from interaction of activity caused by the stimulus with corticocortical inputs. After bipolar derivation, far-field and common reference influences are eliminated and amplitudes decrease, but early and late responses are preserved. **(D)** Example of pairwise phase consistency computed from recording pair of electrodes 5–6 (bipolar) in A1 and electrode 16 in PAF [the 32nd trace in **(A)**]. There is a strong synchronization of activity in the alpha band and the late window, documenting a stimulus-related coupling of these sites.

An example of the original registered activity (before artifact elimination and bipolar derivation) at 6 dB above threshold in both investigated fields is shown in Figure 2. In the individual trials, both in the hearing animal (Figure 2A) and in the CDC (Figure 2B), fast responses following the stimulus within a time window of <100 ms post stimulus (termed *early window* here) can be observed in both fields, although smaller in amplitude and longer in latency in PAF. Approximately 200 ms after the stimulus, a second increase in activity is observable that is less well synchronized (time-locked) with the onset of the stimulus and thus variable from trial to trial, yet is very different from prestimulus activity (>200 ms termed *late window* here). When a single recording contact is considered, the reduced synchronization relative to stimulus onset in the late window becomes apparent (Figure 2C). The synchronized response, predominantly observed in the early window, will be called *evoked response* and the response that is not synchronized, predominantly observed in the late window, will be called *induced response* (for previous detailed analysis, see Yusuf et al., 2017). Using such signals pairwise phase consistency can be computed in a frequency-specific manner (Figure 2D). The peak PPC increases after the stimulus reached values of up to 0.4. In some recording positions, peaks in coupling were more pronounced in the early window.

### Stimulus-Related Connectivity: Congenital Deafness Reduces Top–Down Interactions

We first identified individual simultaneously recorded site-pairs that showed significant couplings at any frequency (*z*-score estimation using jackknife procedure, see section “Materials and Methods”), being around half of all compared electrode pairs in all three groups (ACs: 61%; ECs: 49%; CDCs: 53%). This corresponds to the observation of similar anatomical connectivity between these two cortical areas between deaf and hearing cats (Barone et al., 2013; Butler et al., 2017). Only these coupled site pairs were used for further analysis.

**FIGURE 3:**
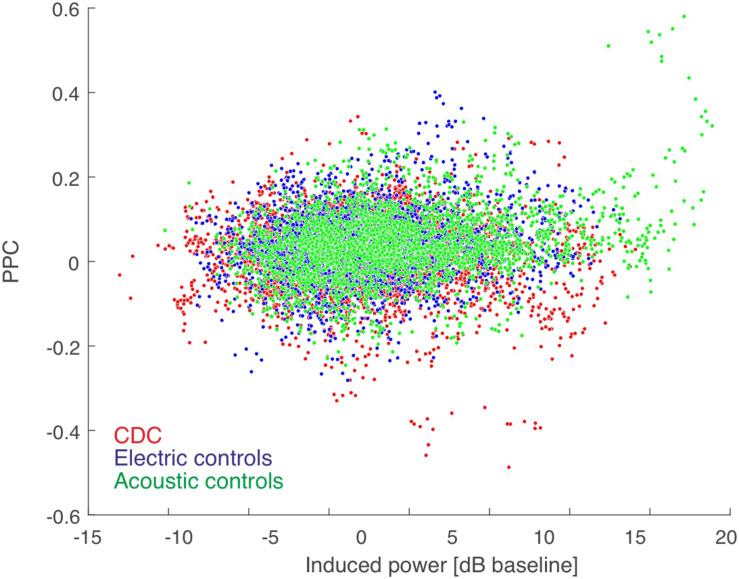
PPC-based connectivity is not a direct consequence of induced power (summed for the pair). Color code shown in the inset. The Spearman correlation coefficient was very low (rho = 0.071 for acoustic controls, rho = 0.017 for electric controls, and rho = 0.002 for CDCs, all *p* < 0.05) and thus induced power contributed minimally to the PPC result. There was no difference between the three groups of animals in power-PPC relation.

Next we confirmed that our measure of functional connectivity, i.e., PPC, is not dependent on response strength. We compared the PPC as a function of the sum of the power of the induced responses at the two corresponding positions in all coupled pairs (Figure 3). The very small correlations show that PPC is not dependent on induced power. This further means that the results of connectivity analysis are (as expected) not the consequence of differences in signal power. Thus, differences in signal power in CDCs compared to ECs, as observed in a previous study (Yusuf et al., 2017), did not determine the coupling results of the present study.

**FIGURE 4:**
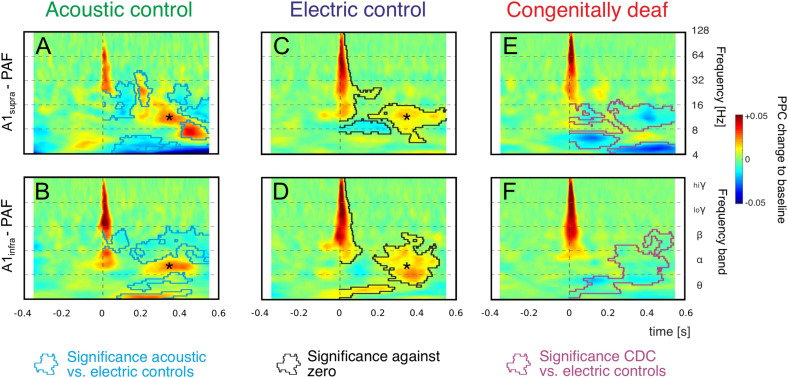
Grand mean averages of time-frequency-based functional connectivity between A1 and PAF computed using pairwise phase consistency (PPC). Functional connectivity computed separately for supragranular layers in A1 (A1_supra_ – PAF, top) and infragranular layers in A1 (A1_infra_ – PAF, bottom) reveal onset responses (near 0 ms) and late responses (>200 ms). Warm (yellow and red) colors represent an increase in synchronization relative to baseline, i.e., a stimulus-related functional connection. **(A,B)**: Acoustic controls: Regions with significant differences to electric controls are outlined with blue lines (non-parametric cluster-based permutation statistical testing, two-tail significant α value = 0.25%). **(C,D)** Electric controls: Regions of significant PPC change to zero are outlined by black lines (comparison to zero, two-tail significant α value = 0.25%), statistical differences to acoustic controls are shown in **(A,B)** as blue lines (non-parametric cluster-based permutation statistical testing, cluster α threshold 0.5%, two-tail significant α value = 0.25%). Alpha band synchronization in the late window (asterisk) is observed in both control groups consistently. **(E,F)** In CDCs, only onset couplings are preserved, all late (>200 ms) couplings disappeared. Regions with significant differences to electric controls are outlined with magenta lines (non-parametric cluster-based permutation statistical testing, two-tail significant α value = 0.25%).

Stimulus-related coupling increases were observed in both the early and late windows in both control groups. Grand mean averages for all three groups investigated are shown in Figure 4. The mean values underestimate the PPC increases observed in individual recording pairs (as in Figure 2D) due to differences in exact timing and frequency between the pairs, but the grand means reflect the most common features of the couplings and are appropriate for robust statistical comparisons.

We concentrated on the increases in PPC relative to baseline, since these reflect stimulus-related functional coupling between the sites. Both acoustic and electric controls, irrespective of the recorded layer, showed an increase in coupling in beta and gamma bands in the early window (Figures 4A–D). Increased coupling was also observed in the alpha band, but this finding was limited to some layer groups only. A second period of alpha coupling appeared in the late window (asterisk, see also Figure 2), discernible in both control groups and both layer groups. These coupling increases were significantly different from zero (shown in Figures 4C,D as black lines, cluster-based permutation test, two-tail significant α = 0.25%). In both control groups we observed also variable desynchronizations in the late window.

Statistical analysis of the differences between the two control groups is shown by the blue lines in Figures 4A,B (cluster-based permutation test, two-tail significant α = 0.25%). In general, the early connectivity as well as the late connectivity (asterisk) were not different. However, smaller “islands” of desynchronization, together with a beta and theta coupling in the late window, were larger in amplitude in ACs. This observation may be related to spontaneous activity from the hearing cochlea.

Consistently, in grand means of both acoustic and electric controls and both layer groups, there was (i) an increase in beta and gamma coupling after the stimulus in the early window and (ii) an increase in alpha coupling in the late window.

In CDCs, only the early couplings were preserved. The early synchronization in beta and gamma band was not different from ECs, but in contrast to ECs the early as well as late alpha synchronization disappeared in CDCs (Figures 4E,F; significance to ECs shown by magenta lines, cluster-based permutation test, two-tail significant α = 0.25%). Thus, CDCs differed greatly from the controls: a part of the early coupling as well as all late coupling between A1 and PAF following an auditory stimulus in the alpha band, consistently found in both controls and all layers, disappeared in CDCs (Figure 4E).

**FIGURE 5:**
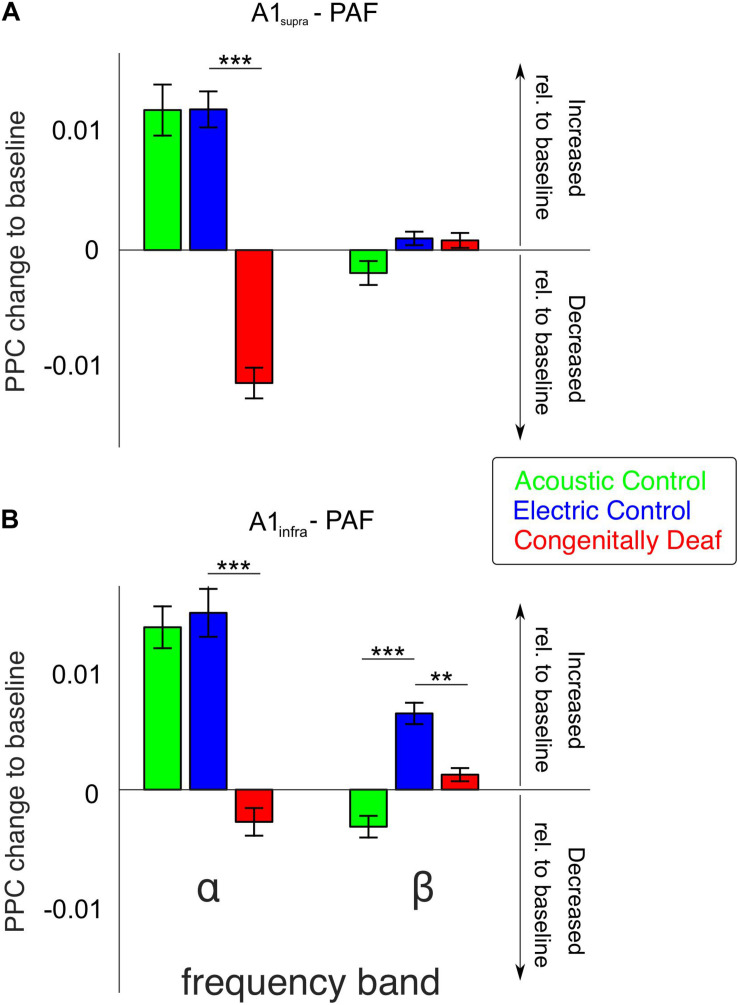
Bar plots of average alpha- and beta-band functional connectivity within the late poststimulus time window for all three groups, separately for A1_supra_ – PAF **(A)** and A1_infra_ – PAF **(B)** couplings. Data shown correspond to the change in PPC relative to baseline (baseline PPC subtracted from poststimulus PPC). Positive (negative) values indicate increased (decreased) functional connectivity relative to pre-stimulus baseline, respectively. Group pairwise comparisons were computed using the two-tailed Wilcoxon rank-sum test. **P < 0.01; ***P < 0.001.

The most extensive effect of developmental experience was observed in the late window. In order to further quantify overall effects we pooled the couplings over the entire late poststimulus time window (>200 ms) in the conventional frequency bands. The theta band, while showing mixed effects in controls and only desynchronizations in CDCs (Figure 4) has to be treated with caution due to the temporal windows available (prestimulus 400 ms) that are at the limit of the temporal requirements for this band, particularly when relative-to-baseline measures are used. Therefore it was not analyzed further. In gamma bands no effects were observed in the late window. Alpha coupling increased following a sensory stimulus in the late window in acoustic and electric controls, whereas it decreased in CDCs (Figure 5; *p* = 3.234 * 10^–17^ for A1_supra_-PAF and *p* = 1.013 * 10^–9^ for A1_infra_-PAF, Wilcoxon rank-sum test). Weaker and less consistent effects were observed in the beta band, where particularly ACs showed the alternating periods of synchronization and desynchronization (Figures 4A,B), leading to a mean desynchronization if summed over time (Figures 5A,B, green bars).

**FIGURE 6:**
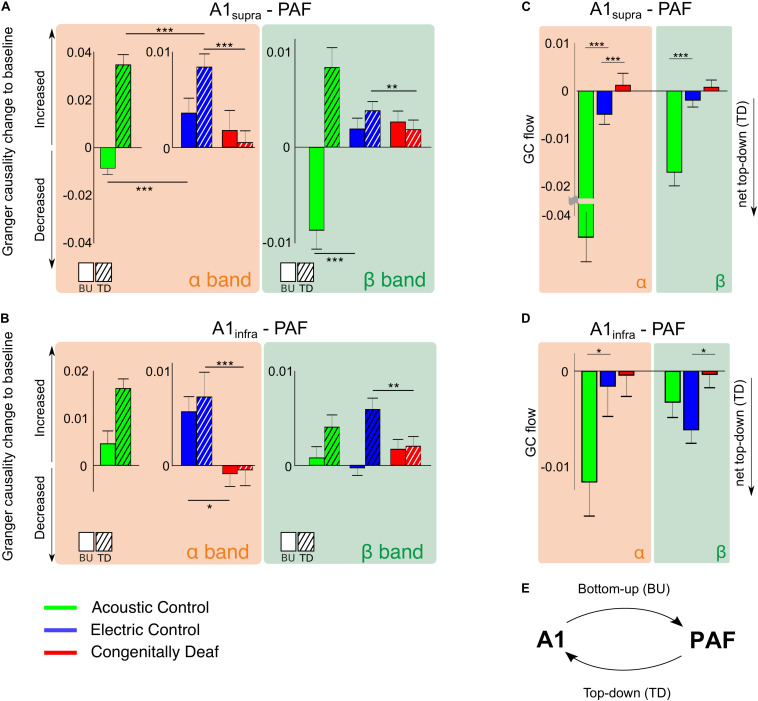
Stimulus-related effective connectivity difference across groups. **(A,B)** Bar plots of alpha and beta band effective connectivity for A1_supra_ – PAF **(A)** and A1_infra_ – PAF **(B)** couplings for all three groups in the late window. Bottom-up (BU) represents connectivity from A1 to PAF while top–down (TD) represents connectivity from PAF to A1. Data shown represent Granger causality (GC) change to baseline (poststimulus GC minus baseline GC). Positive (negative) values indicate increased (decreased) effective connectivity relative to baseline. Solid bars are bottom–up interactions, hatched bars represent top-down interactions. **(C,D)** Barplots of alpha- and beta-band GC flow for A1_supra_ – PAF **(C)** and A1_infra_ – PAF **(D)** couplings for three groups. Data shown are GC flow change to baseline (poststimulus GC flow minus baseline GC flow). GC flow was computed as bottom–up GC minus top–down GC; positive (negative) GC flow values represent domination of bottom–up (top–down) connectivity. Bar plot colors: acoustic control (green), electric control (blue), and congenitally deaf (red) groups. Group pair comparisons were computed using the two-tailed Wilcoxon rank-sum test. *P < 0.05; **P < 0.01; ***P < 0.001. **(E)** Illustration of bottom–up (BU) connectivity from A1 to PAF and top–down (TD) connectivity from PAF to A1.

Granger causality was used to determine the directionality of the alpha and beta A1-PAF interactions in the late window, where PPC differences were found. Both A1 to PAF bottom-up interactions and PAF to A1 top–down interactions were quantified. Previous work has shown that alpha (van Kerkoerle et al., 2014; Michalareas et al., 2016) and beta (Bastos et al., 2015; Michalareas et al., 2016; Richter et al., 2018) bands are associated with stimulus-related top–down feedback between sensory areas. In keeping with these findings, auditory stimulation induced a prominent increase (relative to baseline) of alpha- and beta-band top–down GC in ACs (Figures 6A,B). Also in ECs the increase in top–down GC was larger than the increase in bottom–up GC (Figures 6A,B). Importantly, the CDCs did not show this effect, rather top–down was smaller or same as bottom–up GC, and overall the GC change was small for both supragranular and infragranular layers of A1.

To establish the overall dominant direction of the information flow, we computed the difference between top–down and bottom–up GC, resulting in the Granger flow measure (Fontolan et al., 2014; Babapoor-Farrokhran et al., 2017). Negative Granger flow signifies predominantly top–down-directed interaction, whereas positive Granger flow indicates predominantly bottom–up-directed interaction. The results demonstrate that the stimulus-related change in interaction was a shift toward top–down interaction in both hearing acoustic and electric group (i.e., net top–down interaction). In A1_supra_-PAF coupling, Granger flow in ACs was significantly more negative than in ECs (Figure 6C, *p* = 1.321 * 10^–9^ in the alpha band and *p* = 6.684 * 10^–8^ in the beta band, Wilcoxon rank-sum test).

Granger causality showed weaker top–down connectivity in ECs compared with ACs in all investigated layers of A1 (Figures 6C,D). Additionally, PPC revealed subtle differences in connectivity between acoustic and electric controls (Figure 5). We interpret these observations as a consequence of the artificial electrical stimulus highly synchronizing the auditory nerve firing.

Congenitally deaf cats, on the other hand, lost the top-down flow observed in ECs in the alpha band (Figure 6C, *p* = 4.642 * 10^–4^, Wilcoxon rank-sum test). Beta band, where also ECs showed small effects, was not significant (Figure 6C, *p* = 0.276, Wilcoxon rank-sum test). Remember, in beta band also the PPC outcomes revealed minimal effects (Figure 5). In A1_infra_-PAF coupling, ECs showed stronger net top–down Granger flow in beta-band than CDCs (Figure 6D, *p* = 0.017, Wilcoxon rank-sum test) – in-line with the largest beta connectivity found in PPC in A1_infra_-PAF (Figure 5).

In total, these findings show that the stimulus mode is affecting the connectivity measures. That observation demonstrates that our methods are sensitive to changes in stimulus properties and that functional connectivity can change if the stimulus changes - even in the brain with same anatomic connectivity and same membrane properties of the cortical neurons involved. Absent developmental hearing experience (electric controls vs. congenitally deaf cats) eliminated the stimulus-related coupling increase in the late time window. The results demonstrate that top–down connectivity is substantially involved in the reduced effective connectivity observed in CDCs.

### Ongoing Activity and Connectivity Reveal Layer-Specificity of Deafness Effects

Finally, we tested whether the stimulus-related connectivity could be a mere consequence of resting-state (i.e., ongoing) connectivity. We analyzed ongoing extracellular LFP activities to reveal the power-spectral activity and phase-based connectivity in the absence of an auditory stimulus. It is of importance to emphasize that the groups differed regarding the state of the organ of Corti: whereas ACs had an intact cochlea, in ECs the hair cells were destroyed by intrascalar neomycine injection. Similarly, CDCs did not have surviving hair cells. This is of substantial relevance, because hair cells are the main driver of spontaneous activity in the auditory nerve, providing a tonic drive to the auditory pathway (and auditory cortex; see also discussion). Furthermore, trials containing bursts of activity and spindles were eliminated from the analysis since they may confound connectivity measures (e.g., Valentine and Eggermont, 2001).

**FIGURE 7:**
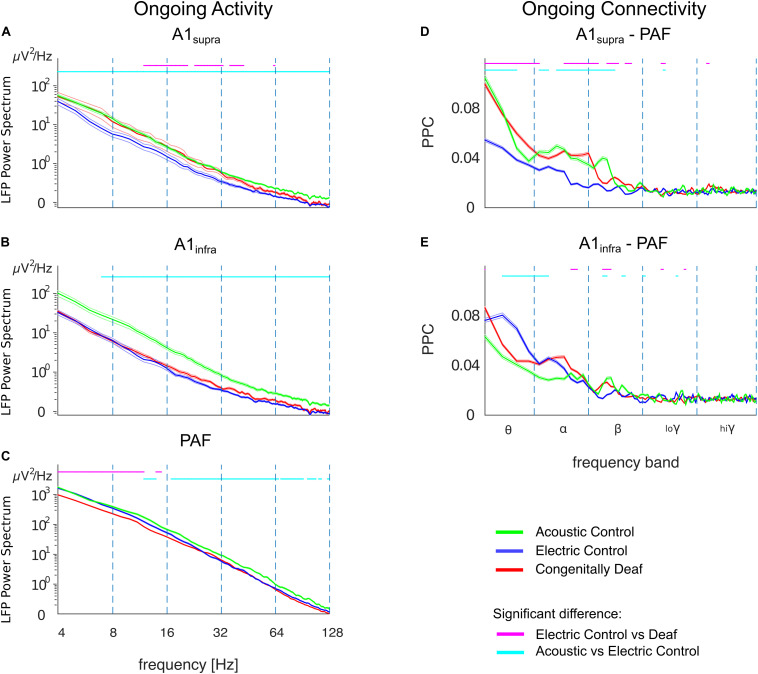
Ongoing LFP power and functional connectivity in A1 and PAF in controls and congenitally deaf cats in absence of stimulation. **(A)** Average LFP power spectra in supragranular A1 (A1_supra_), comparison between acoustic controls with intact cochlea (ACs, green), electric controls with acutely deafened cochlea (ECs, blue), and congenitally deaf cats (CDCs, red) in absence of a stimulus. Data computed from bipolar derivation LFPs. Trials with bursting and spindles were removed before analysis (see section “Materials and Methods”). **(B)** Same as A for infragranular layers in A1 (A1_infra_). **(C)** Average LFP power-spectra in PAF computed from unipolar LFPs. Note the LFP power scale difference. **(D)** A1_supra_ – PAF functional connectivity in absence of stimulation, computed using pairwise phase consistency (PPC). **(E)** Same as D for A1_infra_ – PAF functional connectivity. **(A–E)** Shaded areas represent standard errors of the mean. Statistical pairwise comparisons are shown for electric control vs. deaf (magenta line above the graph) and animals with intact cochleae vs. acutely deafened cochleae (cyan line above the graph) using the two-tailed Wilcoxon rank-sum test (false discovery rate corrected, *p* < 0.001).

The LFP power spectrum revealed a level of ongoing activity significantly higher in ACs than in ECs in almost all frequency bands in A1_supra_, A1_infra_, and PAF (Figures 7A–C). Since these two control groups differ with regard to surviving hair cells and hence spontaneous activity in the auditory nerve, this outcome suggests that, up to the level of the secondary auditory cortex, spontaneous activity in the auditory nerve is possibly a significant factor driving cortical ongoing activity.

The CDCs, in a part of the A1 data, exhibited significantly higher ongoing LFP power than ECs in A1_supra_ for the alpha- and beta-band (Figure 7A, *p* < 0.001, false discovery rate corrected Wilcoxon rank-sum test). This phenomenon was layer specific: it was not observed in the infragranular layers of A1 (Figure 7B, compare anatomical outcomes in Berger et al., 2017). It was also absent in PAF, where the power was in fact significantly lower in CDCs than in ECs in the theta/alpha bands (Figure 7C, *p* < 0.001, false discovery rate corrected Wilcoxon rank-sum test).

The ongoing functional connectivity between A1 and PAF, quantified by the pairwise phase consistency (PPC), also revealed layer-specific differences. There was higher baseline phase coherence between A1_supra_ and PAF in ACs compared with ECs which was significant in theta/alpha/beta bands (Figure 7D; *p* < 0.001, false discovery rate corrected Wilcoxon rank-sum test). This was consistent with the higher ongoing power in ACs, but remember that PPC is power independent (comp. Figure 3). The infragranular layers, despite higher ongoing power in ACs, had a different coupling pattern, with lower baseline coherence between A1_infra_ and PAF in ACs than in ECs; the effect that was most prominent in the theta band (Figure 7E; *p* < 0.001, false discovery rate corrected Wilcoxon rank-sum test).

In congenital deafness, the outcomes differed significantly from ECs particularly for supragranular layers of A1, where CDCs had stronger ongoing coupling to PAF than ECs. This unexpected finding demonstrates that the stimulus-related desynchronization is specific to the auditory stimulus, and further indicates some form of brain adaptation to deafness. Recordings in auditory nerve of CDCs reveal a severely reduced to absent spontaneous activity (Hartmann et al., unpublished observations; for neonatally deafened cats, see Shepherd and Javel, 1997), consequently this rules out a cochlear origin of the difference to ECs.

It is notable that the ongoing and stimulus-related connectivity revealed different outcomes: whereas in stimulus-related connectivity, CDCs showed weakened couplings between PAF and AI with no sign of synchronization increase in the late time window (Figures 4E,F), in ongoing connectivity and supragranular layers they showed a connectivity similar to the ACs (Figure 7D). This dissociation demonstrates that the stimulus-related changes are not a mere consequence of ongoing changes. Furthermore it indicates that CDCs partly compensated the effect of absent ongoing drive from the cochlea.

## Discussion

The present study directly demonstrates reduced functional and effective stimulus-related connectivity following congenital deafness that is specific to the late processing window (>200 ms post stimulus). Particularly top–down interactions were affected by congenital deafness.

In hearing cats, auditory input synchronized the activity between the areas early in the gamma and beta bands and later (>200 ms) in the alpha and (partly) in the beta band (Figure 4). While in CDCs auditory responses were found in both investigated cortical areas (comp. Yusuf et al., 2017), the stimulus-related coupling between them was significantly weakened in the late window. In contrast to controls, the auditory stimulus predominantly caused interareal desynchronization in CDCs (Figures 4E,F). This indicates that the auditory areas of CDCs do not process the stimulus as a functional unit. GC analysis proved that this decoupling mainly reflects reductions in top–down interactions.

The functional and effective connectivity quantifies statistical dependencies between temporal characteristics of neuronal signals (Aertsen and Preissl, 1991; Friston, 2011). Such measures, while faithfully reflecting a functional connection, by definition include the synaptic efficacies of their connections as well as the properties of individual cells and their membranes. All of these affect the ability to form functional connections. The mean evoked LFP response in A1, the consequence of thalamocortical inputs (Lakatos et al., 2009), was not affected by congenital deafness (Kral et al., 2009; Yusuf et al., 2017). This means that the reduced top–down influence from PAF is unlikely due to a downstream effect of a deficient thalamic activation of A1. It might be that bottom-up deficits, either through the weaker A1 → PAF connection, through a weaker thalamic input to PAF, or due to non-reliable responsiveness of PAF neurons to these inputs, could be responsible for the reduced top–down connectivity measures. But in the latter case one would also expect differences in bottom–up connectivity during the early response or in Granger bottom–up results – none of which was the case. The observed reduction of top–down PAF-A1 connectivity was substantially higher than reduction in bottom–up connectivity in the late response in CDCs (Figure 6).

Given these considerations, a reduction in bottom–up drive in PAF neurons in CDCs would not be a sufficient explanation of the drop in top–down connectivity. Furthermore, top–down connectivity systematically exceeded the bottom–up connectivity in both hearing groups (Figure 6), thus the late processing of the stimulus is normally dominated by top–down influences. This was again not the case in deaf cats. While non-linear effects have to be considered, taken together this suggests that the results faithfully reflect a reduced strength of functional connections between A1 and PAF.

Ongoing activity in CDCs in the supragranular (but not infragranular) layers of A1 coupled, on the other hand, more strongly to PAF. This demonstrates that the auditory areas are not generally decoupled in congenitally deaf; rather, they are specifically decoupled during auditory processing.

### Methodology

The approach of accessing fields A1 and PAF and the mapping procedure in A1 in hearing and deaf cats has been validated and described in detail in several previous studies (Kral et al., 2009, 2013; Yusuf et al., 2017). The present results on hearing cats are in line with previous observations of auditory coupling in the auditory cortex of hearing cats, predominantly performed using cross-correlations (Eggermont, 1992, 2000). Previous studies focused on ongoing activity observed a coupling in alpha and beta bands (Eggermont et al., 2011). Auditory correlations increased following an acoustic stimulus (Tomita and Eggermont, 2005), as observed in the present study using phase-based methods (Figure 4). To safely prevent bursting from affecting connectivity (Valentine and Eggermont, 2001), we avoided the burst-suppression state and excluded trials with bursts and spindles (see section “Materials and Methods”). The present study, where comparable in hearing controls, is consistent with previous outcomes in hearing cats.

Since the recording sites in A1 and PAF were >1 cm apart (Figure 1), volume conduction was unlikely contributing to present results. However, to avoid any volume conduction effects, we used bipolar derivation in field A1. Adopting this approach enabled us also to focus on true synchronization between the recorded sites in absence of signals picked up by the reference electrode. Bipolar derivation also provided local signals and allowed layer-specific analysis in A1. Bipolar derivation was applied only in field A1, since (i) it was sufficient to reliably eliminate the influence of volume conduction and common reference on couplings; (ii) penetrations were perpendicular to cortical layers and the electrical homogeneity of the tissue impedance has been previously shown for this direction (review in Mitzdorf, 1985); (iii) the use of unipolar LFPs in PAF had the advantage of capturing signals from a larger number of PAF neurons, also those localized beyond the track direction, increasing the yield and reducing the dependence on the exact recording location within PAF. The possible drawback is a potential overestimation of the absolute overall connectivity.

We analyzed all data using two phase-based connectivity measures. While debiased weighted phase-lag index (WPLId) is insensitive to volume conduction and thus more sensitive for detecting true connectivity than PPC, the results obtained might be exaggerated depending on the phase angle distribution (review in Cohen, 2014) due to a weighting of the imaginary part of the coherence in the WPLId. PPC, on the other hand, is not biased in phase distributions and is also better comparable to previous outcomes of correlational analyses. Therefore, we used WPLId to identify the significantly coupled site pairs and focused on the PPC in order to analyze their coupling strength (Figure 4).

Directionality (effective connectivity) was determined using GC. The results in general corresponded to phase-based measures, but GC additionally showed significant alpha-band difference between AC and EC, which was not observed in PPC. This difference is due to PPC being a symmetric connectivity measure that does not distinguish between bottom–up and top–down influences, considering them aggregately. GC, on the other hand, separated out the information flow and suggested specific decreases and increases in top–down and bottom–up coupling after the stimulus.

To complement the presented coupling analysis with previously used measures of cortical connectivity in hearing cats (Eggermont, 1992, 2000), we additionally performed cross-correlational analysis of the ongoing activity to cross-check our outcomes, obtaining results corresponding to previous studies (results available on request).

Corresponding to previous human data (Hillebrand et al., 2016), also the present study observed a frequency-specificity in the information flow. The alpha band and to an extent (but less consistently) the beta band played a key role in interareal synchronization following an auditory stimulus at late time windows. The present observations support previous findings highlighting the importance of alpha and beta bands in top-down interactions (Buschman and Miller, 2007; van Kerkoerle et al., 2014; Bastos et al., 2015; Michalareas et al., 2016; Richter et al., 2018). In our study, couplings in the gamma band appeared in the early response (within the first 50 ms post stimulus). The gamma band is considered responsible for bottom–up interactions (Fontolan et al., 2014; Bastos et al., 2015) and did not show any significant differences between the groups. However, the early response is additionally strongly affected by thalamic input to both A1 and PAF (Lee and Winer, 2011). We did not observe strong ongoing synchronization in the gamma band in the late window. Previous studies in humans observed ongoing gamma responses during auditory stimulation (Fontolan et al., 2014). In the present study we used very brief stimuli to avoid the interference from electrical stimulation artifacts, an approach validated in several previous papers (Tillein et al., 2010, 2016; Yusuf et al., 2017). This may have reduced such sustained gamma activity. Human studies, on the other hand, typically used long-duration stimuli that may generate more sustained gamma-oscillations (Ray and Maunsell, 2011) due to the early responses that continue throughout the stimulus. Additionally, gamma transients are often coupled to lower-frequency activity (such as alpha) in the late window (Jensen et al., 2014; Yusuf et al., 2017). When using low-impedance electrodes to record from the cortical surface, these transients may combine from several columns and present as sustained oscillations that we observed only as brief transients with recordings from single columns.

### Effects of Anesthesia

Large-scale invasive mapping at dozens of recording positions, including multiple penetrations of the fields, was only possible in anesthetized preparation. The energy of oscillatory phenomena used for coupling quantification is increased by wakefulness, and in particular by attention, but the difference between awake and anesthetized preparations is only quantitative (Fontanini and Katz, 2008; Xing et al., 2012; Sellers et al., 2015), particularly if burst-suppression phenomena are avoided (Land et al., 2012).

Using power-independent measures in the present study eliminated the dependence on signal power, affected by anesthesia. Even when presenting a stimulus passively, it is represented in both primary and secondary fields, and, given this representation, inherently generates both bottom–up and top–down corticocortical interactions, although weaker than in wakefulness and under attention. We obtained significant interareal couplings in both anesthetized control groups. Consequently, while quantitatively stronger coupling can be expected in awake, attentive animals, particularly in top–down interactions (McGinley et al., 2015), and this may yield the statistical comparisons more sensitive, the controls did show significant top–down interactions under anesthesia, and CDCs did not, and the group difference between ECs and CDCs was statistically significant. We can therefore exclude anesthesia as a reason for the differences observed.

### Influence of Stimulus Mode (Acoustic vs. Electric)

Granger causality showed weaker top–down connectivity in ECs compared with ACs in all investigated layers of A1 (Figure 6). We interpret these observations as a consequence of higher synchrony in CI stimulation and the lack of “naturalness” in the electrical stimulus. The reduced interaction in ECs may thus be the consequence of a stimulus that does not fit into the patterns learned throughout life and stored in auditory cortex (Kral and Eggermont, 2007).

The comparison of acoustic and electric controls allows for differentiation of coupling in condition of a known and unknown stimulus. Predictive coding (Friston, 2010; Keller and Mrsic-Flogel, 2018; Vezoli et al., 2020) assumes that the unknown stimulus not fitting into the patterns stored in higher-order areas would generate a strong bottom-up signal (the prediction error). In supragranular layers in ACs, the strong top–down coupling could be interpreted as a strong prediction and the reduced bottom–up coupling the late window (Figure 6A) could be interpreted as a small prediction error. In ECs, the top–down signal (prediction) is smaller and the bottom–up coupling (prediction error) larger. This is consistent with predictive coding. However, in infragranular layers a similar bottom–up signal is observed in both hearing controls. Supragranular layers are the main source of bottom–up stream of information in the cortex, and infragranular layers are the main source of top–down information flow (reviews Hackett, 2011; Markov et al., 2014; Vezoli et al., 2020). That may be the reason why supragranular layers of A1 could better reflect prediction error signaling. However, the present study was not focused on this question and therefore more experiments are required to conclude on this aspect of auditory connectivity.

### Layer Specificity

Ongoing functional connectivity between primary supragranular layers and secondary cortex, on the other hand, was upregulated in A1 of deaf animals to the level observed in hearing animals with functional hair cells (Figure 7D). This may be related to a general increase in the suprathreshold sensitivity of neurons, as observed in the auditory cortex of congenitally deaf animals (Tillein et al., 2010, 2016). This may partly counterbalance the lack of auditory input.

The higher ongoing functional connectivity between A1_supra_ and PAF in CDCs compared to ECs suggests that the supragranular layers tend to developmentally partially compensate the loss of hair cells. Such increased connectivity could be a reason for the increased baseline LFP power in A1_supra_ of CDCs (Figure 7A). In infragranular layers, this phenomenon was not observed, neither in LFP power (Figure 7B) nor in functional connectivity (Figure 7E).

Layer differences are consistent with previous data reporting reduced activity particularly in deep layers of A1 in CDCs (Kral et al., 2006). Deep layers V and VI have specific function in thalamocorticothalamic loops and thus for auditory stimulus conveyed through the thalamus (de Ribaupierre et al., 1972; Steriade, 1999; Derdikman et al., 2003; Castro-Alamancos, 2004). Layer III, on the other hand, is more related to lateral connections to neighboring columns (Rouiller et al., 1991; Markov et al., 2014). Cytoarchitectonic analysis showed that deep layers (but not supragranular layers) are dystrophic in primary and secondary auditory areas of CDCs (Berger et al., 2017). This is consistent with increased ongoing connectivity observed in supragranular layers of CDCs compared to ECs, since higher supragranular coupling in deaf animals may compensate loss of thalamic input by facilitating lateral propagation of activity within the area A1 (Reimer et al., 2011) but also to PAF – potentially related to cross-modal corticocortical reorganization of this field in CDCs (Lomber et al., 2010). Deep layers, on the other hand, are more closely related to the corresponding thalamic nuclei and thus to processing auditory inputs; these demonstrate more auditory-related deficits.

### Development of Corticocortical Connections

**FIGURE 8:**
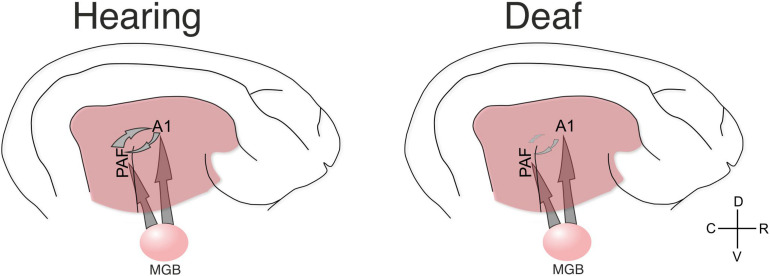
Schematic illustration of the present results. The present data document that in hearing controls the functional connectivity between A1 and PAF is dominated by top–down interactions. Such connectivity is stronger for stimulation that the animal has previous experience with (as shown by comparing acoustic vs. electric stimulation in hearing controls). Both fields additionally receive thalamic input (not directly investigated here) that generates evoked responses and dominates in the early response window. In the deaf animal the functional connectivity in the early window was not significantly different to electric controls, but the late window did not reveal corticocortical synchronization (connectivity) and the dominating influence of top-down information flow disappeared. Yet, responses were observed in both fields, indicating some thalamic input from MGB also in deaf animals (for analysis of the evoked responses, see Yusuf et al., 2017).

Sensory input requires a reciprocal exchange of stimulus-related information at a different level of sensory processing (e.g., different features among each other, or features to object and vice versa) represented in different areas (Malhotra et al., 2004) using interareal couplings (Kral and Sharma, 2012; Kral et al., 2016). This is developmentally shaped by experience that allows for activation, via thalamic inputs, of both primary and secondary auditory areas within a narrow time window of a few milliseconds (Figure 8). Firing within such a time window may strengthen the corticocortical synapses that directly connect these areas by processes of synaptic spike-timing dependent plasticity. A developmental process of this nature functionally defines ‘auditory’ areas by functionally connecting them. Cortical synaptogenesis and synaptic pruning are regulated by hearing experience (Kral et al., 2005). In the absence of auditory input, it may be primarily the synapses that would link different auditory areas during the auditory response that may be excessively pruned (Figure 8).

The anatomical connectome was relatively insensitive to developmental modification of experience and cross-modal reorganization (Kral et al., 2003; Lomber et al., 2010; Barone et al., 2013; Land et al., 2016; Butler et al., 2017). A functional shift in auditory areas toward coupling to the visual system in deaf humans has been demonstrated (Bola et al., 2017). Also this effect was observed at the functional level only. The present data demonstrate that it is the auditory functional connectome itself that is extensively shaped by auditory experience.

Consistent with the different involvement of anatomical and functional connectivity in sensory-related effects, our present findings document a dichotomy in the effect of sensory deprivation on ongoing (partly increased, partly decreased) and stimulus-related (decreased) functional connectivity. Thus resting state connectivity, often analyzed in human imaging (magnetic resonance) studies, cannot be equated with stimulus-related connectivity.

### Consequences for Cochlear-Implanted Subjects

Sensory inputs are constantly embedded in other brain processing and must “fit” to the processes in higher order areas to propagate there (Kral and Eggermont, 2007). If the acoustic stimulus matches such stimulus templates (priors) stored there, it activates the priors in higher regions, and this results in top–down information flow down to the lower areas that interact with the bottom–up stream (e.g., Keller and Mrsic-Flogel, 2018; Schneider et al., 2018; Vezoli et al., 2020). Top–down interactions play a crucial role in filling-in phenomena and extraction of weak signals in a noisy environment (Davis and Johnsrude, 2007; Petkov et al., 2007; Friston, 2010; Riecke et al., 2012; Wild et al., 2012). Top–down interactions have a crucial role also in entrainment to auditory oscillations (Barczak et al., 2018), for speech understanding (Di Liberto et al., 2018) and also for success of cochlear implantation (Zaltz et al., 2020).

The artificial electric stimulus in hearing controls yields significantly weaker top–down connectivity than an acoustic stimulus, most probably since matching with the stimulus priors stored in higher-order areas is poor. At the extreme point, without sensory experience, congenitally deaf animals demonstrate decoupling of connectivity and almost no top–down information flow – likely due to the complete absence of prior internal models. Auditory performance is dependent on bottom–up and top–down interactions during sensory processing (Yusuf et al., 2017), and central processing, executive functioning and “listening strategy” co-determine the benefit of pediatric cochlear implantation (Sharma et al., 2002; Kral et al., 2016). Loss of top–down interactions in the congenitally sensory-deprived brains, normally required for learning control and predictive coding, may be one crucial reason why sensitive periods for therapy of congenital sensory loss eventually close.

## Data Availability Statement

The raw data supporting the conclusions of this article will be made available by the authors, without undue reservation.

## Ethics Statement

The animal study was reviewed and approved by LAVES Oldenburg.

## Author Contributions

AK designed the project and the experiments and obtained the funding. PH, JT, and AK performed the experiments. PY analyzed the data with supervision of PH, AK, and MV. PY drafted the first version of the manuscript, MV and AK edited it. All the authors approved the manuscript.

## Conflict of Interest

JT was employed by MedEl Company, Innsbruck, Austria. The remaining authors declare that the research was conducted in the absence of any commercial or financial relationships that could be construed as a potential conflict of interest.

## Footnotes

1 http://www.ru.nl/fcdonders/fieldtrip/
